# A Disposable Sensor Chip Using a Paste Electrode with Surface-Imprinted Graphite Particles for Rapid and Reagentless Monitoring of Theophylline

**DOI:** 10.3390/molecules27082456

**Published:** 2022-04-11

**Authors:** Aaryashree, Tomoji Ohishi, Yasuo Yoshimi

**Affiliations:** 1 Innovative Global Program, Shibaura Institute of Technology, Tokyo 135-8548, Japan; aarya@sic.shibaura-it.ac.jp; 2 Department of Applied Chemistry, Shibaura Institute of Technology, Tokyo 135-8548, Japan; tooishi@sic.shibaura-it.ac.jp; 3 The Japanese Association of Bio-Intelligence for Well-Being, Saitama 337-8570, Japan

**Keywords:** molecularly imprinted polymer, carbon paste, disposable sensor, theophylline, therapeutic drug monitoring

## Abstract

This work focuses on a carbon-based imprinted polymer composite, employed as a molecular recognition and sensing interface in fabricating a disposable electrochemical sensor. The carbon-paste electrode was made of a molecularly imprinted polymer comprising a copolymer of methacrylic acid as the functional monomer and blended crosslinking monomers of *N*,*N*′-methylenebisacrylamide, and ethylene glycol dimethacrylate, with theophylline as the template. The analytical properties of the proposed theophylline sensor were investigated, and the findings revealed an increase in differential pulse voltammetric current compared to the non-imprinted electrode. Under optimized conditions, the sensor has shown high sensitivity, high selectivity, lower detection limit (2.5 µg/mL), and satisfactory long-term stability. Further, the sensor was tested in whole bovine blood and validated without any matrix effect and cross-reactivity. Additionally, chronoamperometry of the sensor chip supported a rapid determination of THO with a short response time of 3 s. This carbon-paste electrode is highly specific for theophylline and may be applied as a drug sensor for clinical use.

## 1. Introduction

Theophylline (THO) belongs to the xanthine group and has two methyl groups attached at positions 1 and 3. It is the most widely-studied natural xanthine derivative after caffeine and is found in tea, coffee, and cocoa [1]. THO has immense therapeutical importance and is widely used as a bronchodilator [2,3], a muscle relaxant [4,5], a diuretic [6,7], and an anti-asthmatic drug [8,9,10]. Additionally, THO is known as an immunomodulator [11,12], and anti-inflammatory [13,14] and anti-tumor [15,16] agent.

The drawbacks of THO are associated with its narrow therapeutic window (5–15 μg/mL) [17,18] and individually variable pharmacokinetics and pharmacodynamics, along with the repercussion of concomitant medications and diseases [19,20,21]. In addition, a deliberate or accidental overdose of THO may cause high toxicity after a certain period of latency [22,23,24]. The aftermath may include severe conditions such as tachycardia [25], seizures [26,27], excitation in the central nervous system [28], and even death [29,30]. Consequently, therapeutic drug monitoring (TDM) of THO is highly essential. A plethora of techniques has been employed to date for monitoring THO levels, such as various types of chromatography [31,32,33,34], capillary electrophoresis [35], spectrophotometry [36,37], immunoassays [38,39,40], and differential derivative spectroscopy [41,42]. Many of these techniques are very complicated and necessitate time-consuming operations and the expertise of skilled technicians.

Electrochemical techniques have gathered vast interest because of their high sensitivity, relative simplicity, rapid response time, and low cost [17]. The literature review reveals substantial efforts to determine THO using electrochemical methods, achieving detection limits up to 10^−8^ M [4,18,19,20,21]. Most of the electrodes mentioned above, however, have complex fabrication procedures (such as lengthy experimental procedures lasting for several days [2], use of complex electrode modifiers such as liposomes and aptamers [43,44], and use of enhancing agents or surfactants [45]) and are not convenient for real-time use. The most widely used electrodes for studying the electroanalytical activity of THO are carbon-based.

Molecularly imprinted polymers (MIPs) are artificially tailored recognition elements having molecular recognition cavities to replicate the features of natural receptors [46]. MIPs are capable of recognizing and binding to specific target molecules. The molecular imprinting process involves the copolymerization of functional monomers and crosslinking monomers in the presence of the target molecule as a template [47]. The target interacts with the functional monomer to produce a complex during the copolymerization process with crosslinking. The removal of the template from the complex in the copolymer by proper extraction creates cavities that complement the template’s size, shape, and chemical functionality. The MIP technology encompasses several application areas such as sensors, drug delivery, and catalysis [48,49]. The success of MIP formation depends upon the ability of the template to form a complex with the functional monomer during polymerization. Chemical stability plays a crucial role when co-polymerizing a template and a functional monomer. Additionally, it is essential to focus on the crosslinking monomers to have a highly selective MIP. Crosslinking monomers attribute to the flexibility and surface hydrophilicity (especially when MIP and template are supposed to interact in polar solvents), contributing to the ability of the MIP to distinguish between the target and other analogs [50].

Different MIPs with varied synthesis techniques have been reported for several therapeutic drugs in the last decade. However, only a few works in the literature report MIPs for THO for real-time use. In one of our previous studies, an electrochemical sensor based on a MIP grafted carbon paste electrode for THO detection exhibited the capability of measuring the drug over a wide investigation range [48]. This sensor consisted of a ceramic base and platinum wiring connected at the bottom for the three electrodes, i.e., a counter electrode (platinum, dia. 2 mm), a reference electrode (silver/silver chloride ink, dia. 0.9 mm), and a working electrode (MIP, dia. 0.9 mm). This ceramic chip sensor was also shown to be helpful for the detection of other drugs such as vancomycin and meropenem (antibiotics) and phenobarbital (antiepileptic).

This paper reports a paper-based THO sensor with molecularly imprinted graphite as the electrode material. This study involved the optimization of an MIP by blending the crosslinking monomers and the voltammetry parameters for sensitive detection of THO. The sensor was tested on whole bovine blood, and the response was compared to that in buffer saline solution. Additionally, we performed chronoamperometry on the disposable paper chip, obtaining a response in a few seconds.

## 2. Results

### 2.1. Sensitivity

**Figure 1:**
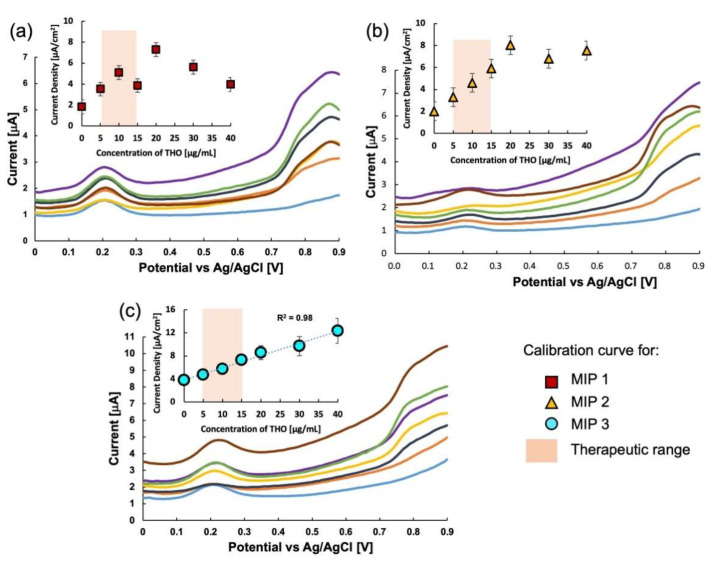
Differential pulse voltammograms showing the response of the three MIP sensors to THO in buffer saline solution of pH 7.4: (**a**) the response of MIP 1 or MBAA-only MIP, (**b**) the response of MIP 2 or EDMA-only MIP, and (**c**) the response of MIP 3 or MBAA+EDMA MIP. The insets of (**a**–**c**) represent the relationship between the current intensity at 0.8 V and THO concentration.

Sensitivity was determined using the differential pulse voltammetry technique. The ferrocene oxidation peak was observed in blank and spiked buffer saline solutions (pH 7.4). To assess the feasibility of this method for the quantitative analysis of THO, the relationship between the redox current as obtained by DPV and the concentration of THO was determined. The response of the three MIPs in the buffer saline solution with variable THO concentrations is shown in Figure 1. MIP 1 (crosslinked by MBAA only) is not specifically responsive to changes in the THO concentration, as is clear from Figure 1a. For concentrations up to 10 μg/mL, the response increased linearly, then it became unstable, and at concentrations ≥ 20 μg/mL, it showed a decreasing response with an increase in the concentration of THO. The response of MIP 2 (crosslinked EDMA only) was also quite linear up to 15 μg/mL, which is the maximum therapeutic blood level for THO. Still, the response was somewhat unstable at concentrations above 15 μg/mL, as shown in Figure 1b. MIP 3 (crosslinker was blended) exhibited a stable voltammogram and the highest linearity between 0–40 μg/mL in Figure 1c. The stable response of MIP 3 throughout the test concentration range of THO and the high linearity indicates that blending of the crosslinking monomers, MBAA and EDMA, is a good strategy for obtaining stable cavities in the MIP, which enables high sensitivity to THO.

The values of the responses of the three MIPs are listed in Table 1. The response for MIP 3 is four times greater than that for MIP 1 and 1.6 times greater than that for MIP 2. Furthermore, the coefficient of correlation (*R*^2^) for MIP 3 was 0.99, indicating a nearly linear response of the sensor.

### 2.2. Contact Angle Measurement

**Figure 2:**
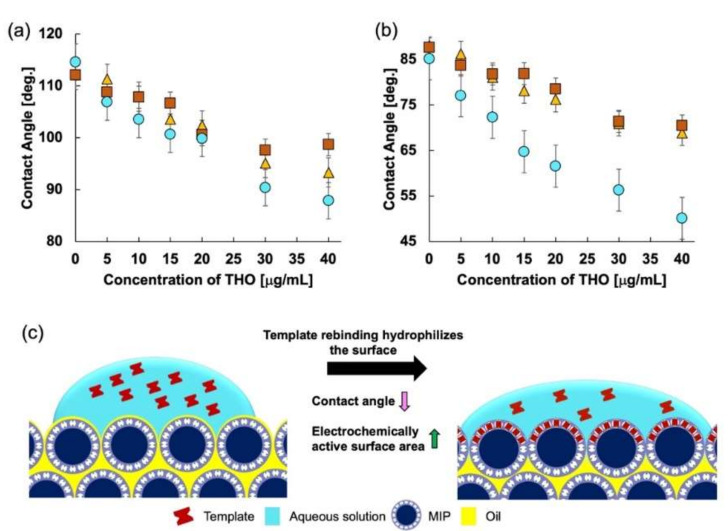
(**a**) Advancing contact angle of the three MIPs, (**b**) receding contact angle of the three MIPs: squares, triangles, and circles represent MIP 1, MIP 2, and MIP 3, respectively, and (**c**) illustration of the decreasing contact angle with increasing MIP rebinding. As the template rebinds with the MIP, the surface is hydrophilized and thus the contact angle decreases.

Contact angle measurements for each MIP were performed to investigate further the reason for such an enhanced response for MIP 3. The sessile drop method was implemented [51] to measure the contact angle. Figure 2 shows the change in contact angle with the change in THO concentration for all three MIPs. The change in contact angle for MIP 3 is the largest, resulting in higher sensitivity than for MIP 1 and MIP 2. The percentage change in the receding contact angles for all the MIPs is listed in Table 1.

MIP 3 has a receding angle change of more than 41%, while MIP 1 and MIP 2 have receding angle changes of slightly more than 21% and 19%, respectively. This increase in the change of contact angle for MIP 3 is almost twice that for the other MIPs, which is also evident from the sensitivity changes shown in Figure 1. More target molecules (THO) occupy the available specific rebinding sites as the concentration increases. The hydrophilic properties of MIP 1 and MIP 2 should be very different. However, the fact that there is little difference in the water contact angle for the electrode surface using either MIP indicates that the contact angle for their surfaces is dominated by the oil contained in the paste electrodes.

The interaction of THO with the binding sites swells the binding sites, thereby pushing the oil back into the bulk of the paste electrode and making the surface more hydrophilic. This increases the area for anodic oxidation of the redox marker ferrocene dissolved in the sample solution [48,52,53,54]. Additionally, the inherent nature of the crosslinking monomers affects the interaction of THO with the binding sites by surface characteristics. The MIP containing EDMA as the crosslinking monomer has a hydrophobic surface, whereas the MIP with MBAA as the crosslinking monomer has a hydrophilic surface. The MIP with the blended crosslinker has a distributed phase of hydrophilic and hydrophobic sites. Therefore, the variation in contact angle at its surface is beneficial for improved sensing. The hydrophobic surface created due to EDMA somewhat inhibits the interaction of the target and the binding sites on the MIP. When only MBAA is used, the hydrophobicity decreases, but due to the flexibility of the crosslinker matrix, the binding is not uniform, and thus unstable current is obtained. However, when the crosslinking monomers are blended, the hydrophilicity of MBAA and the rigidity of EDMA are retained, thus giving a stable and linear response.

### 2.3. Optimization of Scan Rate

**Figure 3:**
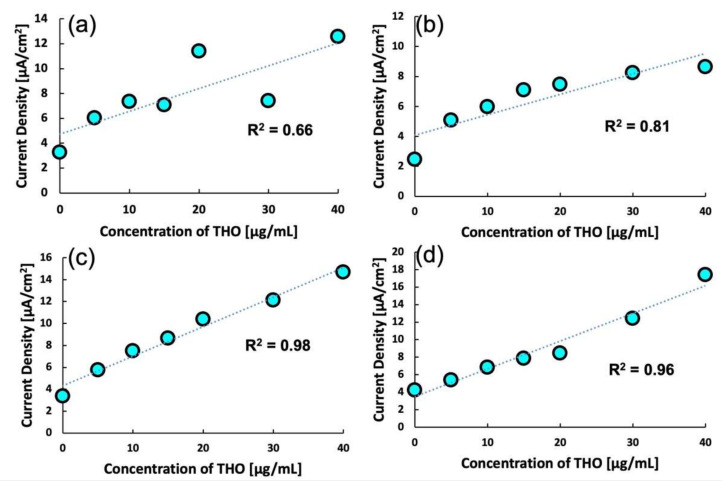
Calibration curves for MIP 3 at various scan rates: (**a**) 5 mV/s, (**b**) 10 mV/s, (**c**) 20 mV/s, and (**d**) 50 mV/s.

Before commencing the analytical studies on the MIP, the scan rate for DPV for MIP 3 was optimized. As shown in Figure 3, four different scan rates were tested to obtain the most suitable value. At scan rates of 5 mV/s and 10 mV/s, the analyte could be detected but at the cost of linearity. However, upon increasing the scan rate to 20 mV/s, a stable and highly linear response was observed. Upon further increasing the scan rate to 50 mV/s, the response remained the same, but the linearity decreased slightly. For every analysis, the pre-treatment time was 10 s at a potential of 0.0 V. The pre-treatment time was essential for obtaining a stable output current. Such a dependency of sensitivity and linearity on the scan rate can be attributed to the surface-phenomena-based sensitivity of the MIP. When the scan rate is lowered, bulk electrochemical activity begins that interferes with the sensitivity of the MIP. Therefore, the scan rate for subsequent experiments was fixed at 20 mV/s.

### 2.4. Confirmation of Enhanced Response after Imprinting

**Figure 4:**
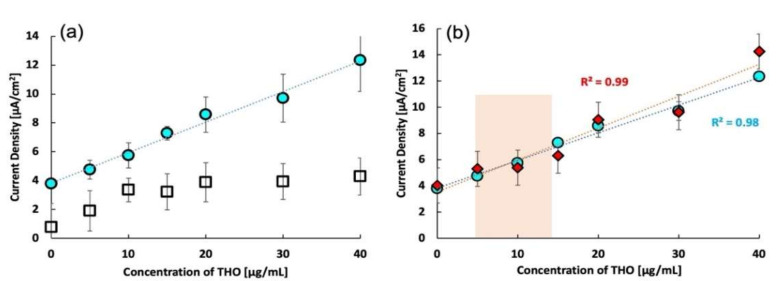
(**a**) Calibration curves of the response of MIP and NIP in buffer saline solution, (**b**) comparison of calibration curves of MIP response to theophylline in buffer saline and whole bovine blood. Circles represent the imprinted polymer response, squares represent the non-imprinted polymer response, and diamonds represent the response of MIP in whole bovine blood.

After optimizing the composition and the scan rate, the effect of imprinting was confirmed for MIP 3. In this control experiment, a non-imprinted polymer (NIP) was synthesized by the same process as that used for MIP 3, except for the omission of the template. The responses of both MIP and NIP are plotted in Figure 4a, which clearly shows the high sensitivity of the MIP sensor over the NIP sensor.

For important analytes, rigorous laboratory-based analytical techniques have been reported [55,56,57]. With this in mind, we recorded the response of the THO sensor in spiked whole bovine blood. Figure 4b shows the calibration curves for the responses of the MIP sensor to THO in buffer saline and whole bovine blood. The responses of both are comparable. The squared correlation coefficient *R*^2^ is larger than 0.98, indicating a linear response. These results suggest that the present THO sensor can be successfully used to monitor THO directly in whole blood without the cumbersome separation of plasma from the blood.

### 2.5. Calibration Stability

**Figure 5:**
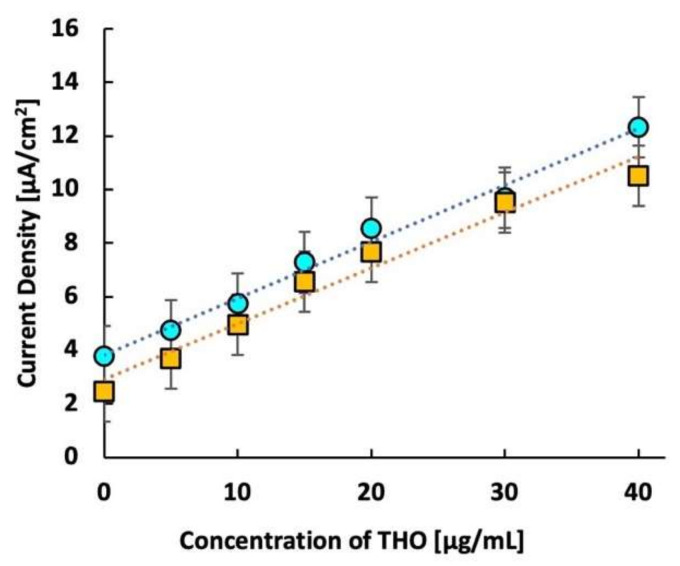
Calibration curve for MIP in buffer saline after 55 days. The circles represent the MIP response on day 1 and the squares represent the response after 55 days of storage at room temperature.

Molecularly-imprinted polymers are generally stable at standard room temperature and pressure. To verify the stability of the synthesized MIP, the MIP was stored under ambient room conditions for several days, and the response to THO was re-recorded. Figure 5 compares the calibration curves for the THO MIP on day 1 and day 55. After 55 days, the sensor’s response remained slightly more than 97% of the initial value. However, the linearity was somewhat reduced (*R*^2^ = 0.97). Therefore, we can confirm that the sensor was relatively stable and that it can be kept at room temperature without any sophisticated ambiance and with very little loss of sensitivity. The reproducibility of the proposed sensor was investigated by comparing the peak current for THO by using four imprinted sensors. Based on the measurements from each sensor, the relative standard deviation (RSD) was found to be approximately 1.2% on average for each concentration.

Additionally, the limit of detection (LOD) and limit of quantification were also calculated and found to be 2.5 μg/mL and 8.2 μg/mL, respectively. Since the LOD < LOQ, it is evident that there could be some difference between the measurement and calculation data. Therefore, to calculate the recovery percentage of the sensor, the concentration value from the best fit curve was calculated for THO concentration of 10 μg/mL. The calculated concentration was 9.008 μg/mL, indicating the recovery to be 90.08%.

### 2.6. Selectivity

**Figure 6:**
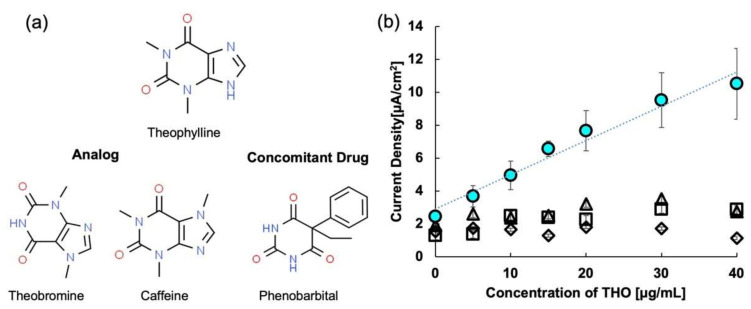
(**a**) Structures of theophylline along with its analogs and concomitant drugs, and (**b**) response of theophylline MIP to analogs and concomitant drugs. Circles represent response to THO. Squares, triangles, and diamonds represent theobromine, caffeine, and phenobarbital, respectively.

Selectivity is the ability to determine how well a sensor can discriminate between the target drug and interfering drugs. The interfering drugs could be analog (structurally similar) or concomitant. In this study, we focused on the selectivity of the THO MIP sensor towards its analogs, caffeine and theobromine, and its concomitant drug, phenobarbital [58,59]. Figure 6 shows the selectivity analysis results for the THO sensor. It is evident from the figure that the MIP sensor is relatively selective for THO. In general, the selectivity of the MIP sensor is determined by the type of crosslinker used. The high selectivity shown by MIP 3 could be due to the correct choice of cross-linking monomer.

### 2.7. Chronoamperometry

Due to its relative simplicity for application and study, chronoamperometry (CA) is a commonly-used approach in electrochemistry [60]. Like most electrochemical processes, CA is extremely sensitive; however, it is not very selective. Electrochemically-active species can diffuse to the surface of the working electrode because of the potential added in an unstirred cell in response to a potential phase perturbation. To balance the change in potential, a large current resulting from the ion flux to the electrode surface gives rise to a capacitive current, which decays rapidly at the start of a potential step (just like any RC circuit). The Faradaic current near the electrode surface decays over time as the mass transfer limit is reached since the concentration of electrochemically active species near the electrode surface decays with increasing distance from the electrode, and the arrival of species at the surface is diffusion constrained. The Cottrell equation describes the exponential decay curve generated by these currents [61,62]:

(1) $$I=\frac{nfA\sqrt{D}c}{\sqrt{\pi t}}$$

where *D* is the diffusion coefficient (/s), *c* is the concentration in a bulk solution (mM), *A* is the surface area of the electrode, *f* is the Faraday constant, *t* is the time (s), and *n* is the number of electrons transferred. From Equation (1), the current is inversely proportional too. This means that the current is high at the beginning for zero initial conditions and gradually decreases with time.

**Figure 7:**
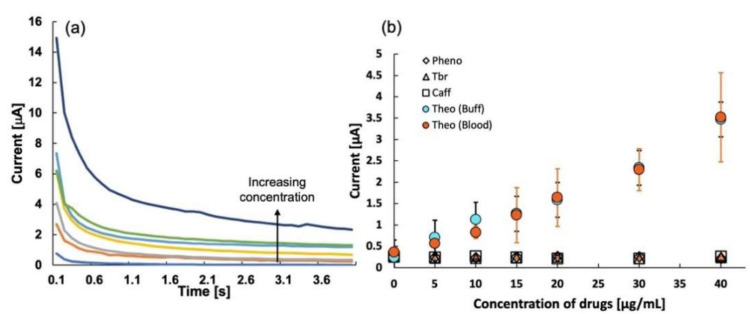
(**a**) Chronoamperometry graph for theophylline sensor, and (**b**) calibration curve for sensitivity of theophylline in buffer and in whole bovine blood. The graph also shows the selectivity of theophylline sensor over theobromine, caffeine, and phenobarbital as obtained from the chronoamperometric analysis.

In our efforts to reduce the testing time for TDM, we performed chronoamperometry measurements on a paper-chip sensor. MIP 3 was used for the trials. For every sample concentration, the single-use technique was implemented. Chronoamperometry was conducted in the high-speed mode for 10 s at a potential of 0.8 V. The calibration curve was plotted using the peak current obtained at 3 s. The chronoamperogram and the calibration curve for the THO sensor are shown in Figure 7 (*n* = 3), which clearly shows a linear correlation between the concentration and the current. The sensitivity and *R*^2^ values for the linear regression were 6.28 and 0.95, respectively. The chronoamperogram seems to follow the Cottrell equation as, initially, the current is huge, but as time increases, electrons generated near the electrode surface (mainly due to rebinding in MIP) are consumed, and the current decreases. The fast oxidation property of ferrocene seems to be an indirect cause of the linear response of the sensor with the increasing THO concentration.

To establish reliable sensing of THO, a selectivity experiment was repeated using the drugs caffeine, theobromine, and phenobarbital. It is clear from Figure 7b that the THO sensor is highly selective, even when using the chronoamperometry method. Not only this but also the sensor’s response in whole bovine blood is as obtained in the DPV analysis, which proves that the sensor can be used for the fast detection of THO. With further optimization of the parameters, the sensitivity and coefficient of linearity can be improved. This indicates a possibility of rapid detection of THO using chronoamperometry, and a sensor such as the commercial blood glucose sensor can be designed with additional material and device parameter optimizations. Thus, our next goal is to develop this disposable sensor further for an ultrafast response to THO and other essential drugs requiring TDM.

## 3. Discussion

The results obtained for the MIP-based THO sensor are entirely satisfactory. The sensitivity of the sensor is good, while the high selectivity and reduced analysis time add to the advantages of this sensor. Equilibrium between hydrophilic and hydrophobic crosslinkers suggests that two crosslinking agents are required to obtain a complete and pure state or network of polymer. The specific interaction at the surface changes by incorporating two different crosslinking monomers having different properties, e.g., hydrophilic, and hydrophobic.

In general, the sensitivity of MIPs is determined by choice of functional monomers [63,64]. However, when measuring in an aqueous medium, the sensitivity is affected by the high polarity of water since water tends to weaken the hydrogen bonds between the MIP and the template. However, surface modification can improve the sensitivity of the MIP. The three MIPs synthesized here had the same amount of functional monomer but different amounts of crosslinking monomers. This suggests that the change in sensitivity of the MIP is due to the crosslinking monomers. The blending of hydrophilic and hydrophobic crosslinking monomers modifies the MIP surface so that a highly hydrophilic region is created on the surface. Thus, MIP sensing in an aqueous medium is facilitated, as is evident from the contact angle measurement data.

Additionally, the blending of crosslinking monomers provides optimum surface flexibility to ensure maximum MIP-template rebinding. As discussed in the contact angle analysis, the oil is pushed from the surface into the bulk when the template rebinds with the MIP cavity and the MIP swells. This phenomenon probably occurs even in the case of electrochemical measurements; therefore, it is considered that the effective area of the electrode surface increases, and the current obtained by the MIP electrode increases. In the case of these three MIPs, it is quite evident that the surface of each of the three MIPs is different.

**Figure 8:**
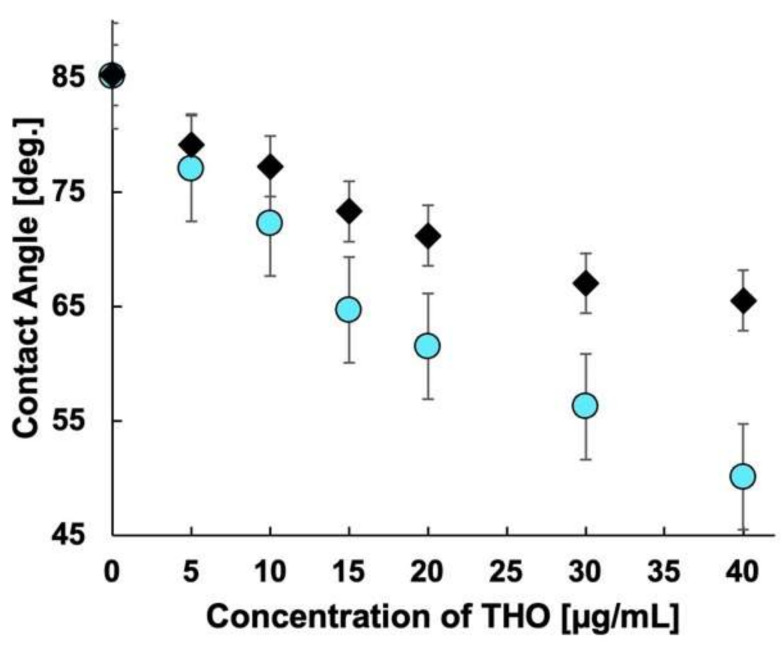
Receding angle measurement for MIP and NIP with change in theophylline concentration. Circle and rhombus show the receding angles for MIP and NIP, respectively.

Compared to the NIP-CP electrode which does not have a molecular identification function, the reduction rate for the contact angle is more than twice as large in the MIP. A comparison of the receding angles for the MIP and NIP is shown in Figure 8. The relative decrease in the contact angle in the NIP was approximately 21%. This suggests that the hydrophilic MIP specifically re-bound with THO swelled, despite hydrophobic silicone oil covering the MIP carbon, which caused a decrease in contact angle. Therefore, it was found that the MIP-CP electrode had a high affinity with THO and that the molecular identification function of the MIP was effective.

Electrochemical sensing relies on the intervention of complex interactions between the template and the imprinted cavity in the MIP against electron transportation between the redox marker and the working electrode to generate signals. The specific interaction between the target molecule (template) and the MIP governs the approachability of the redox marker toward the surface of the base electrode coated with the MIP layer. The faradaic current generated by the redox marker can detect the target drug. This detection technique has been implemented in several of our previous studies [46,65]. However, considering the real-time use of a sensor, the major drawbacks of this technique are: (a) it is very unlikely that a redox marker is naturally present in any body fluid, and (b) adding a redox marker to the sample fluid (especially blood) is a complex procedure. Therefore, we hypothesized that adding ferrocene, an oleophilic redox substance, to the paste electrode’s silicone oil would serve as a redox marker and improve the sensing performance [66]. The details of ferrocene added in silicon oil for various MIPs can be found in our previous study [48]. The ferrocenium ions generated by the anodic reaction readily dissolve in water and hence cannot be used multiple times. Therefore, we have stressed the utility of this sensor as a single-use, disposable sensor. Since the sensor chip is used only once, the loss of ferrocene due to an anodic reaction is not significant enough to produce noteworthy effects.

The chip sensors react similarly in bovine blood and buffer saline solution, which is fascinating. Although a better response can be expected when using plasma alone, using responses close to those in saline is adequate at this stage. Using disposable sensors in a real-time situation is cheaper to reduce operator exposure, infection risks, and research expenses. In addition, most traditional TDM procedures have a run period of 1–2 h since they involve time-consuming operations like blood cell or protein removal. Using differential pulse voltammetry, the current paper-chip sensor can provide results in less than two minutes, allowing physicians and pharmacists to settle on the best timing and volume of drug administration based on real-time drug concentration data. As a result, this single-use sensor can be used to measure the therapeutic effects of THO medications, not only by providing an easy-to-use and bedside measurement tool but also by lowering the cost of analysis since the paper-chip sensor’s overall cost is less than USD 5. A comparison of our chip sensor with commercially available sensors for TDM of theophylline is given in Table 2. These commercial sensors use the spectrophotometric absorbance method to determine theophylline concentration. Although this method is sensitive and accurate, it requires separating blood cells and plasma. This is a complex process and requires expert technicians to obtain the data. Therefore, the paper-chip sensor has the potential to be commercialized for general use for quick and reliable bedside determination of theophylline since it requires no complex processes or expertise.

## 4. Materials and Methods

### 4.1. Chemicals and Other Materials

Methacrylic acid (MAA), *N*,*N′*-methylenebisacrylamide (MBAA), ethylene glycol dimethacrylate (EDMA), phenobarbital sodium salt, caffeine anhydrous, theobromine, and THO were all purchased from Wako Pure Chemical Industry (Osaka, Japan). *N*,*N*-dimethylformamide (DMF), and trisodium citrate dihydrate were purchased from Kanto Chemical Co., Ltd. (Tokyo, Japan). Both MAA and EDMA were distilled under reduced pressure in the presence of a hydroquinone inhibitor. The distilled monomers were stored in the dark at room temperature and in a refrigerator, respectively, before use. Bovine blood for testing was purchased from the Tokyo Shibaura Zoki Corporation (Tokyo, Japan) (5 g/L trisodium citrate dihydrate was added into the blood as an anticoagulant). Spherical graphite particles of diameter 8 µm (SG-BH8), donated by Ito Graphite Co., Ltd. (Kuwana, Japan), were used as the MIP substrate. The conductive ink (LC3111) for the paper was obtained from ePRONICS Co. Ltd. (Tokyo, Japan). Inkjet photo paper (265 µm in thickness and A4 size) was locally purchased from Sanwa Supply Co., Ltd. (Okayama, Japan). Thermally adhesive PET films LZ-A4100 (100 μm) were obtained from Iris Ohyama, Inc. (Sendai, Japan). Silver/silver chloride (Ag/AgCl) ink was purchased from ALS Co., Ltd. (Tokyo).

### 4.2. Instruments

The Fabool CO_2_ Laser by smartDIYs Co., Ltd. (Minami-Alps, Japan) was used to cut the PET sheets. A metal punch with a diameter of 1 mm was obtained from Takagi (Niigata, Japan). Laminator L 409-A (Bonsaii, Dong Guan, China) was used throughout the experiment. The OCA15EC system (DataPhysics Instruments GmbH, Filderstadt, Germany) was used for contact angle measurement. The inkjet printer for the conductive ink (JET circuit inkjet printer) was purchased from ePRONICS Co., Ltd (Tokyo, Japan).

### 4.3. Preparation of MIP

Radical photopolymerization was used for preparing the imprinted polymers. For this, a diethyldithiocarbamate methylene group was added onto the surface of the graphite particles through chloromethylation, as discussed in our previous work [52]. The THO MIP was grafted on the initiator-coated graphite (IG) surface using a general radical polymerization process in the fluidized bed of IG in the solution of monomers and templates. Three different MIPs were made, the compositions of which are tabulated below in Table 3. The three different MIPs were synthesized by dissolving all the constituents of the particular MIP in 10 mL DMF, and radical polymerization and template removal were carried out according to the process in our previous work [46]. The cleaned MIP was dried by vacuum drying. The dried MIP powder was mixed in silicone oil (containing ferrocene) in the ratio of 7:3.

### 4.4. Making the PET Sensor

**Figure 9:**
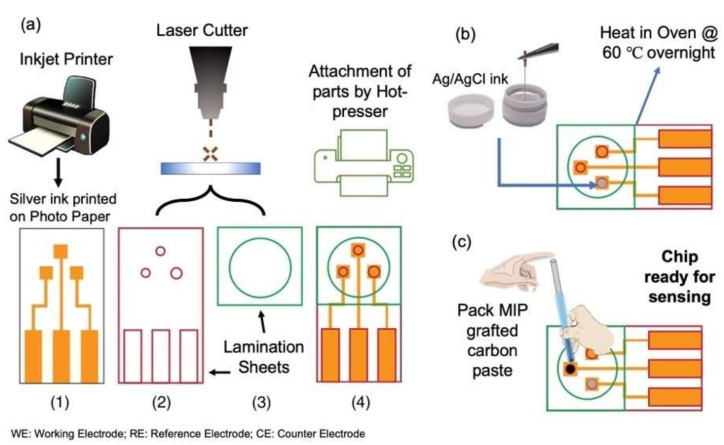
Scheme of the procedure for fabricating the disposable sensor chip. (**a**) Making of chip sensor using paper and PET, (**b**) packing the reference electrode, Ag/AgCl and (**c**) packing the MIP as the working electrode.

The entire process for fabricating the chip sensor is shown schematically in Figure 9**.** In the first step, the wiring film was prepared by printing silver ink using the inkjet printer JET Circuit, specified for conductive ink on photo paper, as seen in Figure 9(a–1). The design for the base electrodes, electrode holes (reference and counter), and reservoir were all made using Adobe Illustrator CC 2020 (institutional license). The circular holes for counter electrodes (2 mm in diameter), reference electrodes (1 mm in diameter), and reservoir (10 mm in diameter) were made by cutting the thermally-adhesive PET films using the CO_2_ laser cutter as in Figure 9(a–2). The working electrodes were then punched using a metal punch with a diameter of 1 mm.

This was done to maintain the uniformity of the working electrode. Next, using a laminator L 409-A, the PET film with the electrode holes was attached to the paper-printed electrodes, and the PET film with the reservoir hole was laminated, as seen in Figure 9(a–3,a–4). Silver/silver chloride (Ag/AgCl) ink (ALS Co., Ltd., Tokyo, Japan) was packed in the reference electrode hole (Figure 9b) and left to dry overnight in the oven at 60 °C. Lastly, the prepared MIP was packed in the working electrode hole using a glass tube as in Figure 9c. To ensure smooth and uniform packing of the working electrode, the surface was pressed and polished with a spatula by applying very slight pressure to prevent the MIP paste from sticking to the spatula.

### 4.5. Sample Preparation

All the trials were performed using phosphate buffer saline containing 0.1 M NaCl and a 0.05 M mixture of potassium dihydrogen phosphate and disodium hydrogen phosphate. Samples of different concentrations were prepared in phosphate buffer saline of pH 7.4 and with whole bovine blood. To make different concentrations in blood, 0.01 mg of THO was dissolved in 0.8 mL of physiological saline (9.0 g of NaCl in 1 L of distilled water), and then 19.2 mL of blood was added. Blood samples with THO concentrations ranging from 0–40 μg/mL were prepared using this spiked blood solution.

### 4.6. Electrochemical Parameters

This work used differential pulse voltammetry (DPV) as the main electrochemical technique to determine various sensor parameters. The potential changes made by short pulses overlapping with a step waveform in DPV improve the differentiation of the faradaic current from the charging current. This makes DPV a more sensitive electrochemical technique than cyclic voltammetry. Electrochemical sensing was carried out using a Compactstat.h potentiostat (Ivium Technologies, Eindhoven, The Netherlands) and IviumSoft version 4.012 [3]. After numerous iterations, the initial electrochemical setup was adjusted to achieve the best results. For all the DPV experiments, the following parameters were fixed: scanning potential range = 0.0 V–0.9 V, pulse time = 10 ms, pulse amplitude = 60 mV, step potential (Estep) = 10 mV, scan rate = 20 mV/s (scan rate optimization is separately discussed in Section 2.3), and a pre-treatment was run at 0.0 V for 10 s. Three sets of data were recorded, and the average value of the three was used for calibration. A single chip was used for each concentration, and the scan was run for one cycle only (IviumSoft v4.1084 has provisions for multiple cycle scans). The calibration was done using the current obtained at 0.8 V for each concentration. The chronoamperometric response of the paper-chip sensor is described in Section 2.7. Chronoamperometry was performed (using the same potentiostat) in the high-speed mode for 10 s, at a potential of 0.8 V. The calibration curve was plotted using the peak current obtained at 3 s. The number of samples (*n*) for chronoamperometry was also 3 for each concentration.

## 5. Conclusions

We developed a novel electrochemical approach to THO’s rapid, sensitive, and specific determination. A molecularly imprinted polymer was prepared using a low-cost and straightforward photopolymerization method on initiator-fixed graphite particles as the paste electrode. The developed sensor exhibited high sensitivity and excellent selectivity towards THO. The sensing ability was exceptional, even for whole bovine blood. Additionally, the possibility of using chronoamperometry to detect THO using the developed MIP sensor was established. The sensing time using the technique was reduced to 3 s. This strategy can prove beneficial for developing a portable THO sensor for quick real-time detection of THO in whole blood and thus marks a milestone in applying electrochemical sensors for efficient and appropriate clinical interventions.

## Figures and Tables

**Table 1:** Response of the three types of sensors.

Electrode	Sensitivity (A.m/g)	Change in Contact Angle (0–40 μg/mL)
MIP 1	4.2	21.8
MIP 2	10.6	19.6
MIP 3	16.8	41.1

**Table 2:** Comparison of our disposable sensor with contemporary theophylline monitoring systems.

Technique	Recognition Element	Detection Method	Reagent Required	Stability	Plasma Separation	Operation Time (Min)	Ref.
Beckman Coulter *	Antibody	Absorbance	Yes	2 weeks	Yes	120	[67]
Nanopia *	Antibody	Absorbance	Yes	NA	Yes	10	[68]
Paper-chip sensor	MIP	Electrochemistry	No	4 Weeks	No	<1	This work

* Data obtained from respective assay-kit manuals. The main technique utilized for calibration is absorbance measurement.

**Table 3:** Comparison of the three different MIPs used in this study.

Component	MIP 1	MIP 2	MIP 3
IG	0.25 g	0.25 g	0.25 g
THO	0.15 g	0.15 g	0.15 g
MBAA	1.58 g	-	0.79 g
EDMA	-	1.58 g	0.79 g
MAA	200 μL	200 μL	200 μL

## Data Availability

Not applicable.

## Sample Availability

Samples of the MIP-imprinted graphite and the sensors are available from the authors upon proper contractual arrangements.
